# Ag Surface and Bulk Segregations in Sputtered ZrCuAlNi Metallic Glass Thin Films

**DOI:** 10.3390/ma15051635

**Published:** 2022-02-22

**Authors:** Michael K. Steinhoff, Damian M. Holzapfel, Soheil Karimi Aghda, Deborah Neuß, Peter J. Pöllmann, Marcus Hans, Daniel Primetzhofer, Jochen M. Schneider, Clio Azina

**Affiliations:** 1 Materials Chemistry, RWTH Aachen University, Kopernikusstraße 10, D-52074 Aachen, Germany; michael.steinhoff@rwth-aachen.de (M.K.S.); holzapfel@mch.rwth-aachen.de (D.M.H.); karimi@mch.rwth-aachen.de (S.K.A.); neuss@mch.rwth-aachen.de (D.N.); poellmann@mch.rwth-aachen.de (P.J.P.); hans@mch.rwth-aachen.de (M.H.); schneider@mch.rwth-aachen.de (J.M.S.); 2 Department of Physics and Astronomy, Uppsala University, Lägerhyddsvägen 1, S-75120 Uppsala, Sweden; daniel.primetzhofer@physics.uu.se

**Keywords:** thin film metallic glass, nanocomposites, segregation, electrical resistivity

## Abstract

We report on the formation of Ag-containing ZrCuAlNi thin film metallic glass (nano)composites by a hybrid direct-current magnetron sputtering and high-power pulsed magnetron sputtering process. The effects of Ag content, substrate temperature and substrate bias potential on the phase formation and morphology of the nanocomposites were investigated. While applying a substrate bias potential did not strongly affect the morphological evolution of the films, the Ag content dictated the size and distribution of Ag surface segregations. The films deposited at low temperatures were characterized by strong surface segregations, formed by coalescence and Ostwald ripening, while the volume of the films remained featureless. At higher deposition temperature, elongated Ag segregations were observed in the bulk and a continuous Ag layer was formed at the surface as a result of thermally enhanced surface diffusion. While microstructural observations have allowed identifying both surface and bulk segregations, an indirect method for detecting the presence of Ag segregations is proposed, by measuring the electrical resistivity of the films.

## 1. Introduction

Nanocomposites are being developed for a variety of applications as they allow combining the properties of two or more constituents. Nanoscale reinforcements, such as nanoparticles (NPs), nanowires, etc., which possess unique physicochemical properties, can provide novel functionalities and/or enhance the properties of the matrix. Metallic reinforcements have been embedded in a variety of matrices, the choice of which often depends on the targeted applications [1,2,3]. In fact, metallic reinforcements are often considered as they are ductile and promote recrystallization in ceramics, which leads to finer microstructures, and in turn, impact the mechanical properties [4,5]. More specifically, Ag has often been employed not only because of its chemical inertness but also because of its biocidal properties [6,7] which make it suitable for bio-related applications.

Several studies have reported the formation of Ag-containing nanocomposites by sputtering. For example, TiSiN:Ag [8] and TiCN:Ag [9] nanocomposites were produced by reactively sputtering from a Ti target which contained either Si and Ag, or just Ag pellets, respectively. Domingues and coworkers followed a similar approach to produce AlN:Ag nanocomposites from an Al target comprising a variable number of Ag pellets [10]. With this approach, the number of Ag pellets influenced the Ag content of the films, however, there is little flexibility over the deposition rate of the films as all metallic constituents are operated in the same conditions. Co-sputtering from independent targets, allows for better control over the deposition rates of each constituent, while also varying Ag contents by adjusting the targets powers [11,12,13].

Regardless of the sputtering approach, common features observed in such films were Ag segregations. The presence of these segregations was influenced by process parameters such as the power applied to the Ag target, and therefore, the Ag content [4,14,15], the substrate temperature [16] as well as the bias potential during deposition [11,15,17,18]. The surface segregations were a result of Ag diffusion towards the surface followed by coalescence. The surface and bulk diffusion of Ag atoms can be enhanced with respect to the process parameters listed above. Additionally, highly energetic processes such as high-power pulsed magnetron sputtering (HPPMS) may also contribute to enhanced surface diffusion [15]. Few studies also reported Ostwald ripening as a mechanism for segregation [15,19]. Cloutier et al. used a hybrid method based on plasma enhanced chemical vapor deposition (PECVD) and very low frequency sputtering, to produce Ag-containing diamond-like carbon (DLC) films. The authors reported the presence of Ag clusters on the surface of the DLC as well as within the bulk. In their case, a bimodal size distribution was detected on the surface which supported the occurrence of Ostwald ripening in tandem with diffusion/coalescence processes [19].

The formation of Ag segregations has been reported for several cases of ceramic matrices [2], owing to the immiscibility and lack of reactivity of Ag and the matrices. However, little information can be found on Ag segregation in metallic matrices. In fact, incorporation of Ag in metallic systems is often in the aim of producing novel metallic glass (MG) compositions, often for antibacterial applications [20,21,22]. While efforts are concentrated on identifying the composition range up to decomposition and/or crystallization [23,24,25], the mechanisms of Ag segregation in thin film MGs have not been widely discussed.

In this work, the surface and bulk segregation of Ag in ZrCuAlNi metallic glass thin films is presented and discussed. The films were obtained by a hybrid direct current magnetron sputtering (DCMS)/HPPMS co-sputtering process. The effects of substrate temperature, bias potential and Ag content on the microstructure of the produced films are presented and the mechanisms responsible for surface and bulk segregation are discussed. Finally, electrical resistivity measurements were carried out in order to verify this method for identifying the presence of bulk segregations.

## 2. Materials and Methods

**Figure 1:**
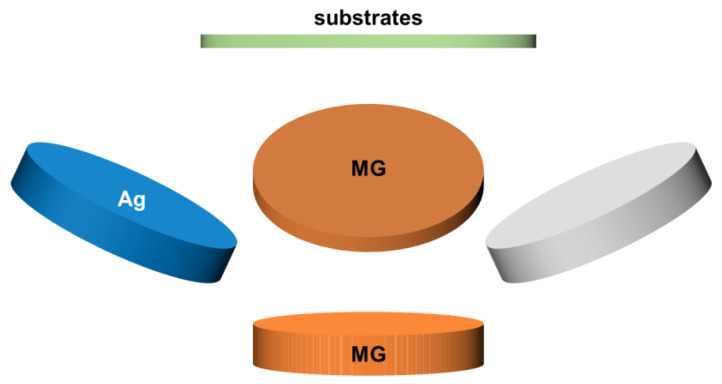
Schematic of target positioning in the ultra-high vacuum (UHV) chamber. The grey disc, on the right-hand side, represents the fourth magnetron of the chamber which was not used during depositions.

Ag-containing metallic glasses were deposited using co-sputtering from an elemental Ag target (Ø 20 mm, 99.99%, M&K GmbH), operated in HPPMS, and two identical Zr_60_Cu_25_Al_10_Ni_5_ powder metallurgical composite targets, which will be referred to as MG in the text (Ø 20 mm, PLANSEE Composite Materials GmbH, Lechbruck Am See, Germany), operated in DCMS. Pure thin film MGs were also deposited, for comparison, in DCMS mode. An ultra-high vacuum (UHV) chamber, with a base pressure below 4 × 10^−6^ Pa and a target-to-substrate distance of 100 mm, was used to deposit the MG and Ag-containing MGs on 10 × 10 mm^2^ c-cut sapphire, Al_2_O_3_(0001) substrates which were used as delivered, i.e., with no pre-cleaning. All targets were operated in constant power mode, while the substrates were rotated to ensure a homogeneous elemental distribution. Ar (99.9999%) was used as sputtering gas with a pressure 0.55 Pa. Figure 1 represents the target positions in the UHV chamber, and more information on the geometry within the chamber can be found elsewhere [26]. HPPMS and DCMS were performed using a Melec SIPP2000USB-10-500-S pulser combined with a 1.5 kW ADL GP 15/1000 DC power supply and an Advanced Converters PMP-1 DC power supply, respectively.

The parameters that were varied during depositions are summarized in Table 1. The Ag content was controlled by changing the applied power on the MG targets, while keeping the Ag one constant. For example, for the high Ag content (30 at.%) the metallic glass targets were operated at 22 W, each. For lower Ag contents, the target power on the metallic glass targets were increased. The powers used were decided after test depositions were carried out to determine the Ag content and deposition rate at each condition. Then, the sputtering times were adapted in order to deposit ~1 μm-thick films. Here too, test depositions were carried out to adapt the deposition durations. Indeed, for 30 at.% Ag, the operating power of the MG targets were decreased, which resulted in lower deposition rates. Therefore, the deposition duration was increased in order to deposit ~1 μm-thick film (cross-sections measured in the focused ion beam microscope).

Structural properties of the deposited films were characterized by means of X-ray diffraction (XRD), using a Bruker AXS D8 Discover X-ray diffractometer with an integrated general area detector diffraction system (GADDS), with Cu radiation. An incident angle of 15° was used, while diffractograms were collected within the 2θ range of 20 to 70°. X-ray reflectivity (XRR) was used to determine the density of the pure MG films. The measurements were carried out in a Panalytical Empyrean diffractometer with Cu radiation and GenX was used for fitting [27].

Ion beam analysis was carried out at the Tandem Laboratory of Uppsala University. Chemical composition depth profiling was done through time-of-flight elastic recoil detection analysis (ERDA). We directed 36 MeV ^127^I^8+^ projectiles at the specimen with incidence and exit angles of 22.5° with respect to the sample surface, while the angle between the ion beam and detector was 45°. Since Cu and Ni cannot be straightforwardly differentiated due to similar mass, their ratio was determined more accurately by Rutherford backscattering spectrometry (RBS) using 2 MeV ^4^He^+^ primary ions detected at a scattering angle of 170°. RBS also further improves the accuracy in the deduced Zr:Ag ratio. A detailed discussion of accuracies achievable in a combination of different ion beam analytical methods for systems based on multiple transition metals can be found elsewhere [28]. ERDA and RBS data analysis was undertaken using CONTES [29] and SIMNRA [30], respectively.

Chemical compositions were measured with a JEOL JSM-6480 scanning electron microscope equipped with an EDAX Genesis 2000 energy-dispersive X-ray spectroscopy (EDX) detector. Measurements were carried out with an acceleration voltage of 7 kV and a magnification of 4000× under a high vacuum. EDX spectra were quantified based on two reference samples which have been measured by ion beam analysis: Zr_50_Cu_26_Al_10_Ni_7_O_7_ was used for determining the Zr-Cu-Al-Ni ratios, while the Ag and O reference concentrations were obtained from a Zr_43_Cu_20_Al_7_Ni_8_Ag_17_O_5_ film. Scanning electron microscopy (SEM) micrographs were captured with a Hitachi TM4000Plus Tabletop microscope using a backscattered electron (BSE) detector.

Thin lamellae and atom probe tomography (APT) specimens were prepared in a FEI Helios NanoLab dual-beam focused ion beam (FIB) microscope, using Ga^+^ ions at 30 kV acceleration voltage. Scanning transmission electron microscopy (STEM) was carried out on the lamellae using a STEM III detector in high-angle annular dark field (HAADF) mode. Furthermore, EDX line scans (quantification without standard) were carried out with an EDAX Octane Elect EDX detector.

Three-dimensional spatially-resolved chemical composition analysis at the nanometer scale was carried out by atom probe tomography (APT) using a CAMECA local electrode atom probe 4000× HR. Laser-assisted evaporation was employed with 50 pJ laser pulse energy, 125 kHz laser pulse frequency, 60 K base temperature and 0.5% average detection rate. Data analysis was undertaken using IVAS 3.8.0.

The resistivity of the films was measured using a Van der Pauw setup containing four contacts which were pressed onto the sample surface. A Keithley 2611B System SourceMeter Unit (SMU) was used to provide the current of 5 mA and to measure the resulting voltage. An average of eight sheet resistance values was obtained for all possible configurations of the four contacts, in order to exclude the effect of contact resistance, and repeated 20 times, resulting in one resistivity value. The procedure was repeated three times by removing the contacts and placing them on the surface again. The electrical resistivity was then deduced by considering the thicknesses of each film. One resistivity datapoint given corresponds to the average of the three measurements and the error bars correspond to the standard deviation.

## 3. Results and Discussion

**Figure 2:**
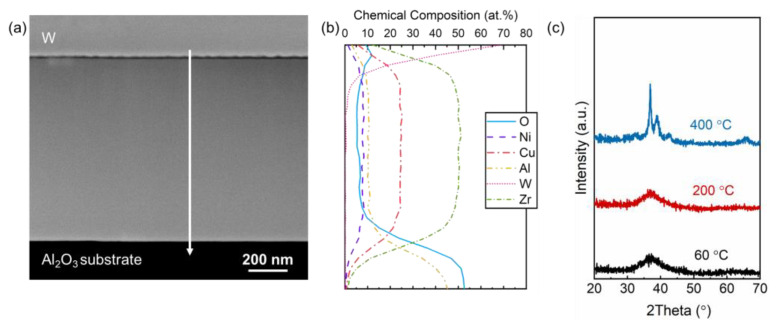
(**a**) High-angle annular dark field scanning transmission electron microscopy (HAADF STEM) image of the ZrCuAlNi film deposited at 200 °C with direct current magnetron sputtering (DCMS) and (**b**) energy-dispersive X-ray spectroscopy (EDX) line scan measured along the arrow represented in (**a**). (**c**) X-ray diffraction of thin-film metallic glasses (TFMGs) deposited at 60, 200 and 400 °C.

Bönninghoff et al. discussed the growth of a Zr_60_Cu_25_Al_10_Ni_5_ thin-film metallic glass (TFMG) produced by HPPMS and DCMS, and have reported differences in the microstructure and density of the films produced [31]. Considering the material system of the pure MG being identical to that presented in this work, albeit from a target produced by a different manufacturer, films were reproduced in DCMS and HPPMS modes in order to confirm that the microstructures produced were featureless. It appeared that both the films deposited at 60 (not shown) and 200 °C, resulted in featureless microstructures, as seen in Figure 2a, regardless of the deposition mode. The density of the film deposited here with DCMS was 6.8 g/cm^3^, which is as dense as the film deposited with HPPMS in [31]. Furthermore, the chemical composition was homogeneous throughout the thickness of the films as determined by ERDA depth profiling and EDX line scans (see Figure 2b). Therefore, the MG targets were operated in DCMS to avoid decreased deposition rates related to the HPPMS mode [32].

Figure 2c gathers the X-ray diffractograms of the TFMGs deposited at 60, 200 and 400 °C. The motivation behind the deposition at a substrate temperature of 60 °C was to avoid possible local heating [15], induced by ion bombardment, during film growth. While the films deposited at 60 and 200 °C did not show any detectable peaks, the films deposited at 400 °C were at least in part crystalline. Therefore, they were not considered further. Furthermore, the films deposited with substrate bias potential were still X-ray amorphous at both 60 and 200 °C, while neither the microstructure nor the elemental distribution appeared to be affected.

**Figure 3:**
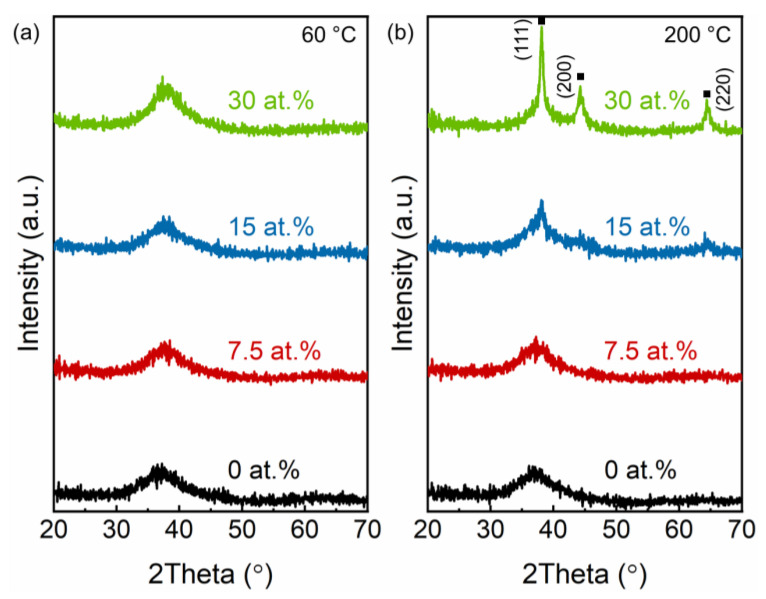
X-ray diffractograms (XRD) of TFMG and Ag-containing TFMGs deposited without substrate bias potential at (**a**) 60 and (**b**) 200 °C.

A hybrid DCMS/HPPMS process was used to deposit the Ag-containing TFMGs in order to promote densification of the films and allow deposition of limited amounts of Ag. The X-ray diffraction patterns of the resulting films deposited without substrate bias at 60 and 200 °C are reported in Figure 3a,b, respectively. While the Ag-containing TFMGs deposited at 60 °C were still X-ray amorphous, one can notice a shift of the broad hump towards higher 2θ angles with increasing Ag content as the films become less Zr-rich. The films containing 15 and 30 at.% of Ag, which were deposited at 200 °C, exhibited peaks corresponding to cubic Ag (ICDD: 00-004-0783). The peaks observed in Figure 3b suggest Ag segregation. While the peaks are of low intensity at 15 at.%, they become more prominent with increasing Ag content, indicating that more Ag has segregated and that the amount of metallic Ag has increased. The XRD patterns of the corresponding films deposited with substrate bias potential (not shown here) were comparable to those deposited without bias. The detection of metallic Ag at high substrate temperature and high Ag contents indicates limited metastable solubility of Ag in the MG, and the simultaneous growth of Ag and MG. The limited solubility of Ag in the MG could be assigned to the positive heat of mixing of Ag and Cu [33,34]. Aside from that binary, Ag-Ni [35] also exhibits a positive heat of mixing while those of the remaining binaries (i.e., Ag-Zr [33] and Ag-Al [36]) are negative. Among the metallic glass constituents, all binaries possess a negative mixing enthalpy [37,38], except Cu-Ni [39]. Therefore, with increasing Ag contents, one may expect Ag segregation as kinetic limitations are overcome by enabling surface diffusion, as well as bulk diffusion due to positive enthalpy of mixing of Ag with two out of four MG constituents.

**Figure 4:**
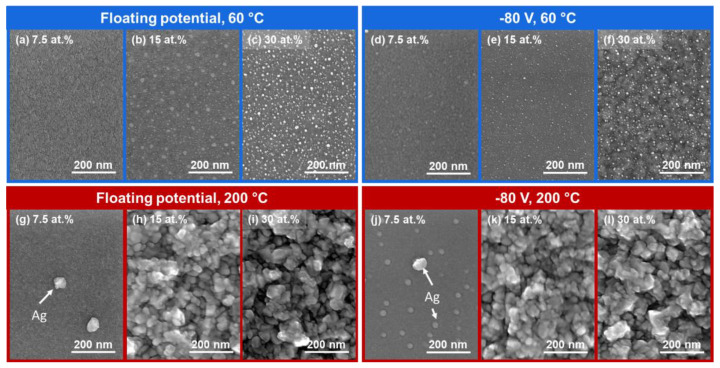
Scanning electron microscopy (SEM) surface micrographs of films of different Ag contents deposited at (**a**–**c**) floating potential; 60 °C, (**d**–**f**) −80 V; 60 °C, (**g**–**i**) floating potential; 200 °C, and (**j**–**l**) −80 V; 200 °C.

The effect of the substrate bias potential was observed on the surface morphologies of the Ag-containing TFMGs. On the one hand, the 60 °C depositions under floating potential showed the formation of nanoparticles (NPs) at the surface of the films containing more than 15 at.% of Ag (Figure 4a–c). Indeed, at 15 at.% one can observe a bimodal size distribution where the larger NPs are ~42 nm, while the smaller ones are ~25 nm. The size distribution becomes somewhat homogeneous (~35 nm) as the NPs become more numerous when the Ag content rises to 30 at.%. On the other hand, −80 V bias potential led to the formation of NPs already at 7.5 at.% suggesting that the mobility of Ag atoms increased with bias (Figure 4d). The NP size for 7.5 at.% of Ag averaged at ~43 nm and then decreased with increasing Ag content to ~20 nm for 15 at.% of Ag. At 30 at.% two observations could be made. First, the size distribution of the NP was bimodal with the largest NPs averaging ~22 nm, while the smallest ones averaged ~10 nm. Second, an increase in substrate bias potential caused an increase in surface roughness as shown in Figure 4f.

The NP formation is attributed to the low metastable solubility of Ag in the MG, causing Ag to migrate to the surface and to grow initially by coalescence and then by Ostwald ripening. Indeed, the NP size distribution, in Figure 4b in particular, is not homogeneous but rather composed of larger NPs surrounded by smaller ones. These observations are, according to Cloutier et al. [19] and Mulligan et al. [40], consistent with Ostwald ripening. Additionally, it is interesting to note that the unbiased samples with 15 and 30 at.% Ag, deposited at 60 °C were covered with NPs without additional energy contributions, indicating that segregation is mostly caused by higher Ag concentration, as supported by [18].

When comparing the 7.5 at.% Ag-containing films deposited at 200 °C with and without bias (Figure 4g,j), one can clearly see that the bias-induced ion bombardment tends to provide sufficient energy for Ag to migrate and to segregate at the surface. The large grains detected are faceted, similar to what Mulligan et al. observed after annealing CrN:Ag at temperatures comprised between 425 and 625 °C in [40]. With increasing Ag content, a continuous, polycrystalline Ag-rich layer is formed on top of the MG (Figure 4h,i,k,l). This layer is partly responsible for the Ag peaks indexed in Figure 3b. The formation of such layer is also due to the enhanced mobility of Ag atoms, provided by the increased substrate temperature combined with the slower deposition rates that accompany the high Ag-content depositions. Indeed, longer depositions times allowed for Ag to diffuse, segregate and form the Ag layer. Similar observations were made by Hu et al. who reported the heat-induced formation of a dense Ag layer on the coating surface of a YSZ:Ag-Mo nanocomposite coating [41].

**Figure 5:**
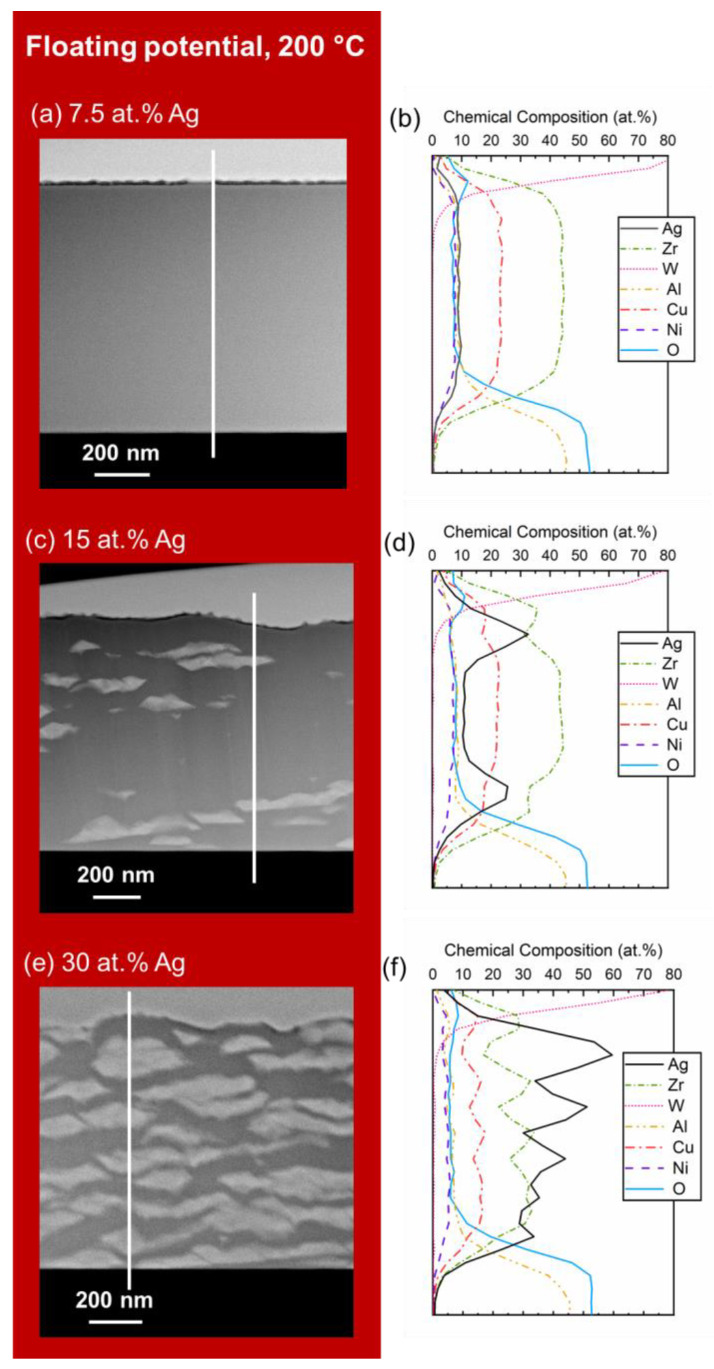
STEM cross-sectional HAADF images and corresponding EDX line scans of the films deposited at 200 °C, under floating potential, containing (**a**,**b**) 7.5, (**c**,**d**) 15, and (**e**,**f**) 30 at.% Ag.

While Ag was detected at the surface of most films either in the form of NPs or in the form of polycrystalline layers, the presence of Ag segregations throughout the film thickness remained a possibility. Therefore, cross-section lamellae of films were examined by STEM in HAADF mode, while the compositions along the film thicknesses were collected using EDX (Figure 5). The films deposited at 60 °C, of all Ag contents, with and without bias exhibited the same featureless cross-section microstructure and homogeneous composition as the film shown in Figure 5a. The lack of features, therefore, supports the preferential surface segregation at lower substrate temperatures, regardless of Ag content. However, the Ag-containing films with ≥15 at.% of Ag deposited at 200 °C were characterized by continuous Ag layers on the surface (similar thicknesses for both Ag contents) and Ag segregations in the volume of the TFMG, confirming the formation of a dual-phase composite film of Ag (light grey) embedded in a featureless MG matrix (dark grey). Indeed, these films exhibited elongated Ag agglomerates in the volume of the TFMGs (Figure 5c,e) and the size and number increased with increasing Ag content. This finding is similar to observations made by Mulligan et al. and Incerti et al. in the case of CrN:Ag nanocomposites [40,42], according to whom the lamellar shape can be attributed to continuous growth on top of the elongated agglomerates. As shown from the line scans in Figure 5d,f, these agglomerates are Ag-based, indicating that they could also contribute to the Ag peaks indexed in the XRD pattern in Figure 3. Therefore, there seems to be a threshold Ag content which is needed to start the agglomeration within the bulk of the film at higher temperatures. The formation of the Ag segregations was independent of bias potential, indicating that it was directly related to the substrate temperature. In fact, as mentioned previously the substrate temperature is responsible for the enhanced surface diffusion of Ag species.

The overall compositions of the produced films are provided in Table 2. The compositions are fairly comparable regardless of deposition temperature or substrate bias potential. Significant changes occur when varying the Ag content. With increasing Ag content, Cu and Zr appear to decrease the most. At 30 at.% Ag all MG constituents decrease as the Zr to Ag ratio approaches 1. The films were characterized by ~3 to 9 at.% O.

**Figure 6:**
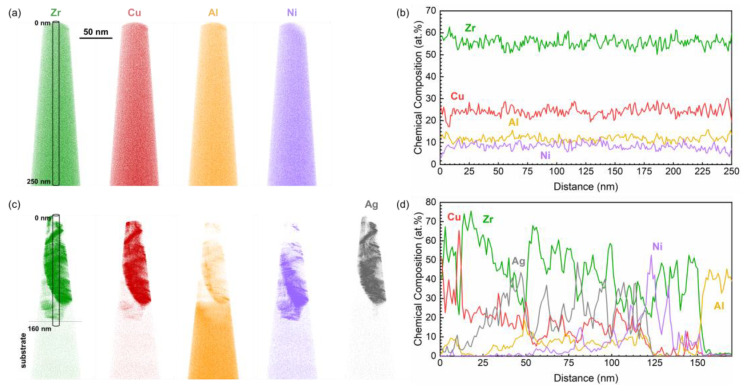
(**a**) Elemental reconstruction of Zr, Cu, Al, and Ni positions, and (**b**) concentration profile of the cylindrical region indicated in (**a**), of the pure TFMG, deposited without bias at 60 °C. (**c**) Reconstruction of Zr, Cu, Al, Ni, and Ag positions, and (**d**) concentration profile of the cylindrical region indicated in (**c**), of the TFMG containing 30 at.% Ag, deposited without bias at 60 °C.

In order to examine the effect of non-segregated Ag on the elemental distribution of the MG deposited at low temperature, APT was performed on specimens retrieved from unbiased TFMGs deposited at 60 °C, without and with 30 at.% Ag. The reconstructions of the atomic positions and the concentration profiles corresponding to each film are shown in Figure 6. The chemical composition, at the nanometer scale, of the pure MG is homogeneous and characterized by a random elemental distribution, as seen in Figure 6a. Furthermore, the concentration profile in Figure 6b is representative of the composition of the target material (oxygen content < 0.3 at.%). The insertion of Ag has a non-negligible effect on the elemental distribution in the metallic glass. Indeed, segregations can be seen in Figure 6c, while the concentration profiles of all elements, in Figure 6d, fluctuate significantly. The fluctuations are in agreement with observations made by Oh et al. as one may notice that in the Ag-depleted regions, the Cu concentration is higher [43]. More importantly, in some of these regions, the Cu concentration exceeds the nominal 25 at.% of the original MG, indicating regions of Cu clustering within the film. The distribution of Zr is also significantly affected and seems to fluctuate in a similar manner as Cu, although further investigations would be required to conclude on its behavior. Although present in lower contents, the Al and Ni profiles are also impacted by the Ag addition. It is important to note that the film considered with 30 at.% Ag is actually no longer Zr-rich, as the Zr to Ag ratio is close to 1, according to EDX measurements in Table 2 and supported by the broad band shift observed in Figure 3a. Therefore, we suggest the formation of a new Ag- and Zr-rich MG whose elemental distribution is inhomogeneous.

Ag has been known to change the local structure of Cu-containing metallic glasses because of the positive heat of mixing of Ag and Cu [33,34]. However, these changes seem to also depend on the Ag concentration. Gammer et al. reported that even small concentration variations may affect the medium range order of CuZrAl(Ag) metallic glasses. In fact, they showed that Ag concentrations >5 at.% destabilize the B2 structured clusters [44]. Furthermore, Rajan et al. did not observe any phase separation in the ZrCuAlAg TFMG they produced (~5 at.% in Ag) [45]. Therefore, one may expect similar inhomogeneities in elemental distribution as presented in Figure 6 for Ag contents exceeding 5 at.%.

**Figure 7:**
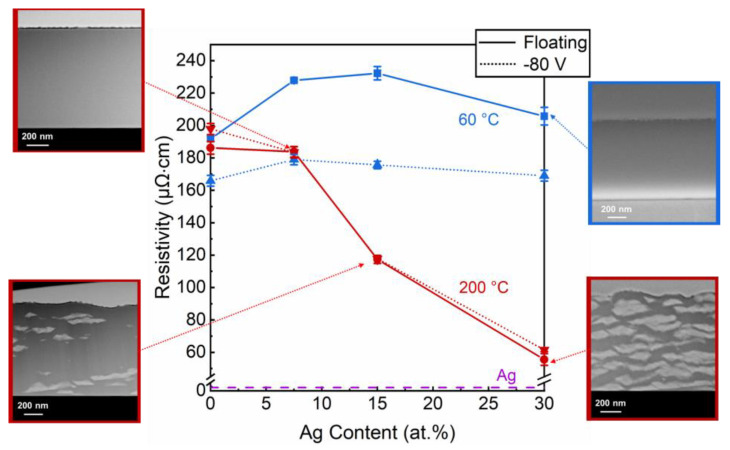
Electrical resistivities of films deposited with (continuous lines) and without (dotted lines) bias at 60 (blue) and 200 °C (red), for the different Ag contents, accompanied by corresponding cross-section SEM (30 at.% Ag, floating, 60 °C) and STEM images (7.5–30 at.% Ag, floating, 200 °C).

The electrical resistivities of all films were calculated from measured sheet resistances and thicknesses, and are plotted in Figure 7 as a function of Ag content. STEM and SEM cross-section micrographs of selected samples are also provided. Ag and Cu exhibit the lowest electrical resistivities among the metals considered herein (Table 3). As the Ag content is increasing, and the Zr decreasing, the formation of Ag segregations should be evidenced by a drop in the electrical resistivity of the film.

As can be seen from Figure 7, this is true in the case of the films deposited at 200 °C, with and without bias, in which the formation of elongated Ag clusters was observed in cross-sectional images. The resistivity was at its lowest for the 30 at.% Ag films because of the large volume fraction of Ag segregations.

Despite the presence of Ag segregations on the surface of the films deposited at 60 °C, the resistivity marginally increased with increasing Ag content. The fact that, due to lack of thermal activation, no Ag segregates can form in the bulk of the film may be responsible for the increased resistivity. This is observed for all studied Ag concentrations. Additionally, the films deposited at a substrate bias potential of −80 V displayed lower electrical resistivities which may be due to ion bombardment induced densification.

Furthermore, we can conclude that the surface Ag segregations are not affecting the measured resistivities significantly as long as the percolation threshold is not reached. Indeed, films deposited at 60 °C and the low Ag-content films deposited at 200 °C, exhibit discontinuous Ag segregations on the surface. Depositions at 200 °C and Ag contents >15 at.%, led to the formation of a continuous polycrystalline Ag layer on the surface. Considering that the thicknesses of the formed layers are similar for both 15 and 30 at.% Ag, the decrease of the resistivity with increasing Ag content is primarily caused by Ag segregations in the bulk of the film.

Previously, electrical resistivity has been employed to track phase transitions within Cr_2_AlC [46] and the oxidation behavior of TiN and V_2_AlC [47,48]. In this work, we show for the first time, that the formation of Ag segregations in sputtered ZrCuAlNi metallic glass thin films can be tracked by resistivity measurements.

## 4. Conclusions

Ag-containing ZrCuAlNi TFMGs were obtained by hybrid co-sputtering from a ZrCuAlNi target and an elemental Ag target operated in DCMS and HPPMS, respectively. The Ag contents were 0, 7.5, 15, and 30 at.%, and obtained by varying the ZrCuAlNi target power. Substrate bias potential and substrate temperature variations were employed in order to determine their effect on the morphology evolution of the films. Despite the chemical affinity between metals and the amorphous nature of the matrix, the segregation mechanisms appear to be similar to those encountered in ceramics matrices.

Most Ag-containing films deposited at 60 °C exhibited strong surface segregation as Ag NPs were detected on the surfaces of all films except on that of the 7.5 at.%-containing film deposited without bias. Here scanning electron microscopy image analysis revealed the absence of Ag segregations suggesting a critical metastable Ag solubility of >7.5 at.% at this growth condition. The substrate bias potential influenced the surface mobility of incoming Ag atoms, as evidenced by the formation of surface segregations at 7.5 at.% Ag. Increased Ag-contents (≥15 at.%) led to the formation of larger and more numerous Ag segregations, driven by coalescence and Ostwald ripening. Substrate temperature also had an important role in enhancing surface diffusion and promoting coalescence of Ag at the surface, as continuous polycrystalline layers were observed on the surface of the films deposited at 200 °C.

Elongated Ag segregations were observed in the bulk of the films deposited at 200 °C, which contained 15 and 30 at.% of Ag, as a result of thermally enhanced surface diffusion. The MG matrix was still X-ray amorphous and did not present other microstructural features. The formation of metallic Ag segregations observed by cross-sectional microscopy caused a significant decrease in electrical resistivity. In fact, the electrical resistivity was shown to be a useful tool for identifying the presence of conductive Ag segregations in the bulk of the films.

## Figures and Tables

**Table 1:** Deposition parameters used to deposit the pure metallic glasses (MGs) and the Ag-containing thin films.

Deposition Parameters	Zr60Cu25Al10Ni5	Ag
Sputtering mode	DCMS *	HPPMS **
Target power (W)	22–100/target	40
Duty cycle (%)		0.25
t_on_/t_off_		80/31,920
Peak current density (A/cm^2^)		0.18–0.35
Substrate temperature (°C)	60, 200, 400	
Substrate bias potential (V)	Floating ^1^, −80	

* Direct-current magnetron sputtering; ** High power pulsed magnetron sputtering; ^1^ Floating voltage of ~10 V.

**Table 2:** Chemical composition of all films measured by EDX (calibrated with ion beam analysis data).

Sample	Zr (at.%)	Cu (at.%)	Al (at.%)	Ni (at.%)	Ag (at.%)	O (at.%)
0 at.%, floating, 60 °C	52.4	25.4	11.3	7.1	-	3.8
7.5 at.%, floating, 60 °C	50.1	23.1	10.1	6.4	7.7	2.6
15 at.%, floating, 60 °C	45.9	20.7	9.0	5.8	15.1	3.5
30 at.%, floating, 60 °C	38.0	15.4	6.6	4.0	27.5	8.5
0 at.%, floating, 200 °C	53.8	24.8	11.0	6.8	-	3.6
7.5 at.%, floating, 200 °C	49.6	23.0	9.5	5.9	8.0	4.0
15 at.%, floating, 200 °C	46.2	20.0	8.4	5.1	15.6	4.7
30 at.%, floating, 200 °C	36.9	15.3	6.6	4.0	32.6	4.6
0 at.%, −80 V, 60 °C	55.1	25.0	10.1	6.8	-	3.0
7.5 at.%, −80 V, 60 °C	50.6	22.5	8.6	5.9	8.4	4.0
15 at.%, −80 V, 60 °C	46.5	19.8	7.4	4.3	16.1	4.9
30 at.%, −80 V, 60 °C	37.1	14.8	6.1	3.7	32.1	6.2
0 at.%, −80 V, 200 °C	53.7	24.8	11.1	6.6	-	3.8
7.5 at.%, −80 V, 200 °C	52.2	22.9	8.9	6.1	5.7	4.2
15 at.%, −80 V, 200 °C	46.7	19.6	7.5	5.2	16.9	4.1
30 at.%, −80 V, 200 °C	38.7	15.8	6.8	4.1	29.6	5.0

**Table 3:** Electrical resistivities of metals of interest at 20 °C.

Metal	Zr	Cu	Al	Ni	Ag
Electrical resistivity (μΩ·cm)	42	1.7	2.6	7	1.6

## Data Availability

The data that support the findings of this study are available from the corresponding author, CA, upon request.
